# Controlled electromechanical cell stimulation on-a-chip

**DOI:** 10.1038/srep11800

**Published:** 2015-07-02

**Authors:** Andrea Pavesi, Giulia Adriani, Marco Rasponi, Ioannis K. Zervantonakis, Gianfranco B. Fiore, Roger D. Kamm

**Affiliations:** 1 Biosym IRG, Singapore-MIT Alliance for Research and Technology, Singapore; 2 Department of Electronics, Information and Bioengineering, Politecnico di Milano, Milano, Italy; 3 Department of Cell Biology, Harvard Medical School, USA; 4 Department of Biological Engineering and Department of Mechanical Engineering, Massachusetts Institute of Technology, USA

## Abstract

Stem cell research has yielded promising advances in regenerative medicine, but standard assays generally lack the ability to combine different cell stimulations with rapid sample processing and precise fluid control. In this work, we describe the design and fabrication of a micro-scale cell stimulator capable of simultaneously providing mechanical, electrical, and biochemical stimulation, and subsequently extracting detailed morphological and gene-expression analysis on the cellular response. This micro-device offers the opportunity to overcome previous limitations and recreate critical elements of the *in vivo* microenvironment in order to investigate cellular responses to three different stimulations. The platform was validated in experiments using human bone marrow mesenchymal stem cells. These experiments demonstrated the ability for inducing changes in cell morphology, cytoskeletal fiber orientation and changes in gene expression under physiological stimuli. This novel bioengineering approach can be readily applied to various studies, especially in the fields of stem cell biology and regenerative medicine.

Stem cell biology has become a major research focus, but conventional culture systems are often limited in their ability to control local cellular microenvironments and spatiotemporal signaling. Recent studies have reported that mechanical stimulation influences the cell microenvironment and drives stem cell differentiation processes^1^. In parallel, electrical stimulation appears to be equally crucial for the development of conductive and contractile properties of cardiac tissue constructs, as extensively studied by Vunjak-Novakovic and colleagues^2^. Additionally, the simultaneous application of electrical, mechanical and chemical stimuli is required to fully reproduce *in vitro* the native microenvironment of striated muscle *in vivo*^3^. Specifically, for cardiac muscle, the *in vitro* system should be engineered with multiple stimulations to approach the *in vivo* condition in cardiac tissue where the electrical and mechanical signals are strongly coupled^2^.

Consequently, the capability to reproduce *in vitro* the complex native microenvironment combining these simulations, may offer the opportunity to investigate the role of each stimulation to delineate the individual or synergistic effects on the development, function, differentiation or regeneration of the tissue.

Previous studies combining multiple stimulations in a single platform mainly consist of bioreactors at the macroscale^4^,^5^,^6^,^7^,^8^. While these systems provided useful insights into electromechanical phenomena, they require large numbers of cells, large volumes of reagents, and are limited in their accessibility for high resolution and/or time-lapse imaging.

Therefore, the lack of advanced micro-tools to replicate fundamental aspects of the *in vivo* microenvironment (cardiac or skeletal muscle) in a highly controlled manner, including mechanical and electrical stimulation, represents a limiting factor in understanding the causal relationships between single or combined stimulations and their related electrophysiological and morphological consequences^9^,^10^. Specifically, we focused on mimicking the microenvironment of cardiac muscle tissue.

Recent advances in microfluidic technologies have created the possibility of producing *in vitro* assays that provide a range of stimulation capabilities, as well as enabling extensive quantitative assessment of their effects in cells^11^. Microfluidic tools are able to provide defined spatiotemporal conditions with user-controlled input to cells, minimizing differences between *in vitro* models and complex *in vivo* microenvironments^12^. Micro-sized systems can also reduce experimental costs and increase throughput compared to standard cell culture dishes, thus offering a valid alternative to costly and time-consuming animal models.

Most current microfluidic systems, however, are limited to a single mode of stimulation. Regarding mechanical stimulation, these might include mechanical strain, fluid shear stress, and variations in substrate stiffness or nanotopographical features. Examples of mechanical stimulation include that produced by (i) cell stretching using flexible substrates^13^,^14^, (ii) shear forces by generating fluid flow over the cell layer^15^, and (iii) presentation of micro- or nano-patterned features with variable size, geometry, and chemistry^16^. In the case of electrical stimulation, systems that incorporate electrodes for directly applying currents to cells have been developed^17^,^18^. Examples of these systems include electrical stimulation applied to wound healing^19^, regenerative medicine^20^, and stem cell differentiation into cardiac tissue^21^,^22^,^23^. Despite these technological advances in microfluidic tools for stem cell differentiation, the need still exists for micro-devices capable of high-throughput, cost-effective physiological data acquisition with multimodal stimulation^24^.

Here, we report the design, fabrication and validation of a new micro-scale cell stimulator capable of providing simultaneous mechanical, electrical, and biochemical stimulation required for stem cell differentiation studies. The micro-bioreactor was designed to concurrently (i) perform mechanical stretching on a cell culture substrate, (ii) apply a uniform electric field in the cell culture region, and (iii) enable the straightforward delivery of biochemical stimulation. The device also faciliates quantitative measurements of the subsequent effects of each form of stimulation, by using standard equipment found in many biological laboratories. Therefore, the ability to conduct a large number of low-cost experiments under accurately controlled conditions makes this device an appealing tool for pluripotent cell differentiation studies. To test the capacity of our system in controlling key variables for efficient and reproducible electromechanical stimulation, human bone marrow mesenchymal stem cells (hMSCs) were used, which can be differentiated into various types of tissue cells, such as bone, adipose, cartilage, and muscle^25^,^26^. Furthermore, the device can be employed to test cell-based therapies for tissue regeneration, or can be fine-tuned in order to represent various disease models.

## Results

### Device design and fabrication

**Figure 1:**
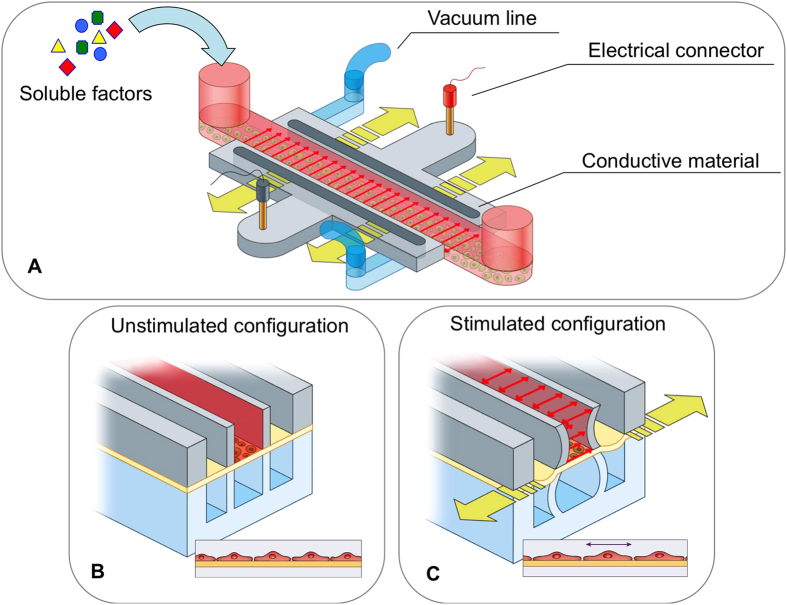
Design of the microfluidic platform developed to investigate the biological cell responses to various stimuli. **A**) Schematic view of the device for applying electrical, mechanical and chemical stimulations. The central channel (in red) is the media channel to provide nutrients and soluble factors to cells. The pneumatic channels (in light blue) perform mechanical stimulation by stretching the PDMS membrane (yellow arrows) where the cells are cultured. The electrical layer contains two conductive regions composed of a mixture of CNTs and PDMS (in light gray), which are connected to the stimulator through two external gold-coated connectors (in red and black). The uniform electric field across the cell culture region is represented by the red arrows. (**B**) Cross section of the device in the unstimulated configuration. **(C**) Cross section of the device in the electromechanical stimulated configuration. Applying vacuum in the two lateral pneumatic channels (in light blue) allows stretching of the cells on the deformable membrane (in yellow).

Polydimethylsiloxane (PDMS) was chosen as the main material for the production of the micro-bioreactor (Fig. 1) due to its favorable features in cell culture applications (namely gas permeability and optical transparency) and the robustness of soft-lithography techniques. In addition, its elastic mechanical properties were exploited to apply controlled strains to cells, while the ability to embed electrical paths by locally doping the PDMS pre-polymer with carbon nanotubes (CNTs)^17^ was used to deliver electrical stimulation.

**Figure 2:**
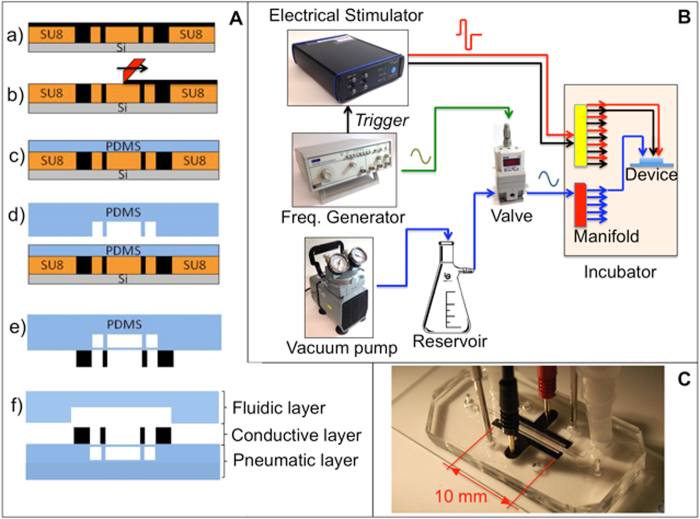
Device fabrication and control system setup. (**A**) Device fabrication steps. (a) Casting of conductive mixture (PDMS + multiwalled CNTs) on top of the silicon wafer mold; **b**) scraping the excess material from the wafer surface and temperature curing; (c) spin coating the 100 μm PDMS layer to obtain a deformable membrane; (d) bonding the pneumatic layer on top of the membrane; (e) demolding the assembled pneumatic and conductive layers; (f) bonding the cover layer in order to seal the fluidic channels. Dimensions are not drawn to scale. (**B**) Schematic of the control system. Alternated vacuum is obtained through an electronic valve controlled by a frequency generator which has the function to trigger the electrical stimulator as well. Inside the incubator, the vacuum and electrical lines are split to provide stimulation to multiple devices in parallel. (**C**) Photo of the final assembled device, including electrical connections and tubing for vacuum control.

The assembly process of the micro-bioreactor is schematically depicted in Fig. 2A(a–f). The device comprises three main layers, namely a pneumatic layer for mechanical stimulation, a conductive layer for electrical simulation, and a fluidic layer for cell culture. We produced the fluidic and pneumatic layers of the PDMS device by standard soft lithography (Fig. 2A). The conductive layer was produced by casting a mixture of CNTs and PDMS on the silicon wafer mold.

When designing the microfluidic system, care was taken to enable precise control over multiple stimulation factors and to ensure compatibility with standard fluorescence and confocal microscopes, in terms of transparency and specimen thickness (to minimize focal plane distance). The PDMS device was designed with two identical chambers (1.2 mm wide, 15 mm long, and 250 μm high), arranged vertically and separated by a thin (~100 μm) membrane (Fig. 1B–C). A design approach similar to the one adopted in the lung-on-a-chip device by Huh *et al.*^27^ was used, in which the thin lateral walls deform when negative pressure is applied to the side channels, thus transmitting a highly uniform strain to the membrane where cells are adhered. Furthermore, both lateral walls of the top chamber are made of a conductive polymer, so that electrical stimulation can be provided to the cells, in addition to the mechanical stimulation. Lastly, biochemical factors can be introduced by pipetting solutions into the inlet of the central channel (Fig. 1A).

Each device that was produced was tested to verify its functionality by adding phosphate buffer saline (PBS) solution to the fluid channel and connecting the device to the vacuum line and electrical connectors. Two tubes were attached to the vacuum access ports by interference fit, and two gold-coated pins were inserted into the electrode access holes (Fig. 2C). The hydraulic tightness was assessed by visual inspection, and a leakage was evident when air bubbles formed in the PBS during mechanical actuation. Electrical connections were tested through measurements, within the solution, with an oscilloscope probe.

The stimulation setup, consisting of a vacuum pump, an electronic valve, an electrical stimulator, and a frequency generator, was located outside the incubator. Only one tube for the vacuum line was required, along with two metallic wires for insertion into the incubator, which was connected to a manifold in order to control multiple devices at the same time. This had the benefit of minimizing deviations from normal cell culture procedures.

### Finite element modeling (FEM)

Finite element simulations were conducted to test the actuation principle of the device and to determine the expected strain field profiles at different pressures. The objective of the simulation was to identify a configuration capable of attaining deformation values from 0% to 8% (to mimic physiological conditions^28^), while minimizing delays that occurs by trial and error method. Efforts were concentrated into finding a simplified production strategy, in order to avoid the additional step required in the lung-on-a-chip microdevice^13^, i.e. the chemical etching of the side membranes resulting from the soft lithography procedure.

The overall system was considered as a 2-dimensional (2D) model, and the device model was comprised of PDMS, whose mechanical behavior was simulated differently depending on the expected deformation regime^29^; thus, the PDMS was incompressible and linearly elastic for small deformations, and hyper-elastic when larger deformations were involved (Supplementary Fig. 1). A static pressure of −700 mmHg was applied to the inner surfaces of the vacuum compartment (orange in Supplementary Fig. 1).

The cell culture membrane attained the desired strain level, even near the chamber sidewalls, while the resulting curvature was minimal (corresponding to a maximum deflection of 14 μm, perpendicular to the initial plane of the membrane). Therefore, we chose to produce the devices without etching the membranes in the side channels where the vacuum was applied, simplifying the production procedure and increasing the production efficiency of the chip.

### Device characterization

**Figure 3:**
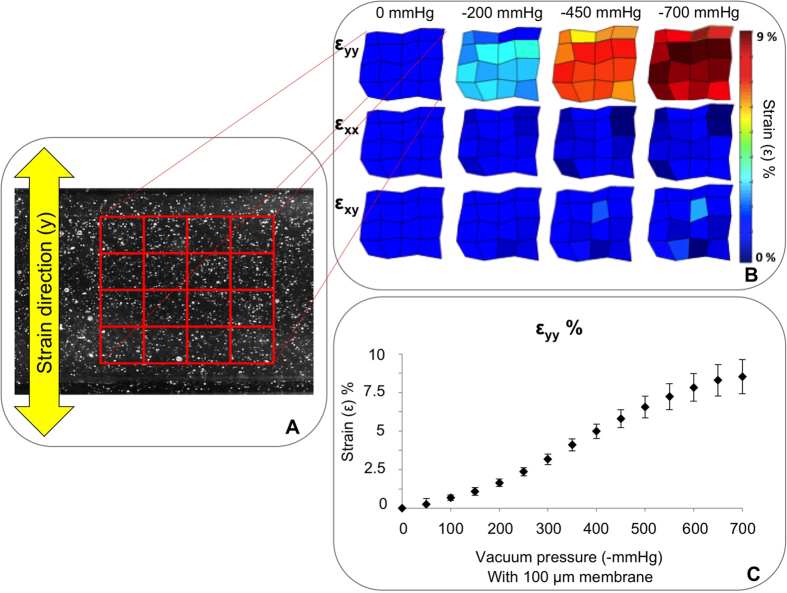
Mechanical characterization of the device. (**A**) Example of a particle-loaded membrane used for the device characterization. A matrix of 5 × 5 points of reference was tracked during the mechanical stimulation. (**B**) Strain map at different vacuum levels with the identified matrix. ε_yy_, ε_xx,_ and ε_xy_ are strains in the direction perpendicular to the channel, parallel to the channel, and shear strain, respectively. (**C**) Calibration curve showing vacuum level versus the resulting strain values. Data are expressed as means ± SD.

Membrane deformation-mediated mechanical stimulation of cells was achieved by controlling the time-dependent air pressure in the two lateral channels flanking the central region (Fig. 1C). The characterization of membrane strain in the device was conducted by tracking displacements of 1μm diameter iron particles embedded in the spin-coated PDMS membranes (Fig. 3). Actuation pressures were produced using an eccentric diaphragm pump, and varied from 0 mmHg to −700 mmHg in 50 mmHg incremental steps (Fig. 3C). By combining a custom-written MATLAB code and a particle tracker ImageJ plug-in^30^, a deformation heat map was constructed, which recorded the position of 25 speckles as they moved through time (Fig. 3A,B).

Color maps were obtained by plotting normal (ε_YY_, ε_XX_) and shear strains (ε_XY_) computed at the nodes of the matrix array (Fig. 3A,B). Measurements of deformation (Fig. 3B) revealed an increased normal strain perpendicular to the fluid channel (y direction) with increasing negative pressures. Conversely, there were no significant deviations in normal strain in the x direction (parallel to the cell channel) nor in shear strain, ε_XY._ These results were obtained with a spin-coated membrane of ~100 μm thickness and, in this condition, an 8% maximum strain was measured at −700 mmHg, consistent with the numerical characterization. A calibration curve obtained by measuring strain corresponding to vacuum pressure levels is shown in Fig. 3C.

The devices were operated for 7 days at 2 Hz to demonstrate their stable strain application. Performing this long-term run did not significantly affect the strain values, showing insignificant levels of material plasticity or fatigue, as supported by a previous long-term study of PDMS pneumatically-actuated elements^31^.

To evaluate the electrical stimulation, a stainless steel needle was inserted into the device through the top layer in order to position its tip at the center of the cell culture region. The needle was connected to a digital oscilloscope and used as a voltage probe. Ultimately, the efficacy of the electrical stimulator was confirmed by measuring the wave signal (−1.2 V for 1 ms, −1.2 V for 1 ms, 1 Hz) during electrical stimulation of the device to have an electric field of 5 V/cm.

### Cell orientation and immunofluorescence staining

Cell stretching experiments were performed using hMSCs (Lonza Group Ltd., Basel Switzerland), and the effects of the electromechanical stimulation on this cell line were analyzed. After a 24 h static culture, a 3% or 7% strain at 1 Hz was applied to the membrane to which the cells were adherent, as reported in the literature for similar applications^32^,^33^. For electrical stimulation, a biphasic signal was chosen, as used in previous studies^20^,^23^. Specifically, in order to stimulate the cells with an average electric field of 5 V/cm, biphasic square-wave pulses (+1.2 V for 1 ms, −1.2 V for 1 ms, 1 Hz) were applied.

**Figure 4:**
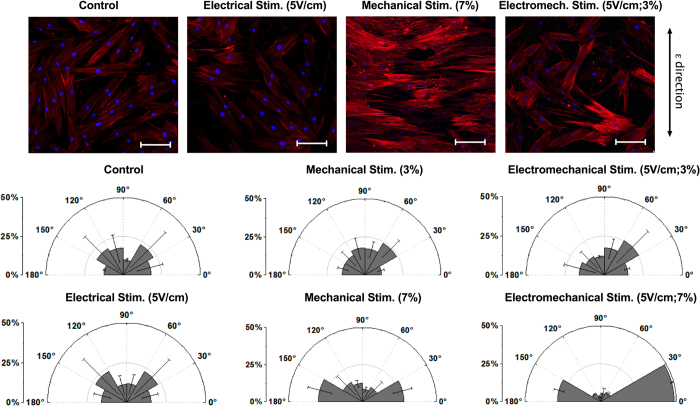
Qualitative and quantitative cell orientation analysis under mechanical stimulation (3% and 7% strain), electrical stimulation (5 V/cm), electromechanical stimulation (3% strain and 5 V/cm or 7% strain and 5 V/cm), and control condition (no stimulation). **(A**) Nuclear and actin cytoskeleton staining. The strain direction is indicated by the arrow on the right. Scale bar is 100μm. (**B**) Percentage of cytoskeletal structures oriented in a given direction from 0° (horizontal, perpendicular to strain direction) to ± 90° (vertical, parallel to strain direction). Data are expressed as means ± SD.

Cell orientation analysis was performed after 14 days of stimulation using the Fiji directionality plugin^34^, based on Fourier spectra analysis. To evaluate the orientation distribution of cytoskeleton fibers, results from five devices per configuration (namely *control*, *mechanical*, *electrical and electromechanical*) were analyzed by plotting means and standard deviations (SD). Orientation data were compared using a t-test with p values <0.05 considered as statistically significant. Morphological changes in cells subjected to mechanical, electrical or electromechanical stimulation were compared to control cells that were not exposed to any stimulation (Fig. 4A). Interestingly, the actin fibers in mechanically-stimulated cells were oriented perpendicular to the strain direction and showed a more elongated spindle-like morphology compared to control cells. These findings are consistent with previous observation of cytoskeletal reorganization of MSCs and endothelial cells under shear stress stimulation^35^,^36^. Orientations were quantified (Fig. 4B) by setting 0° as the cell orientation perpendicular to the strain direction and 90° as that parallel to the strain direction. In the cases of mechanical or electromechanical stimulation with 3% strain, or electrical stimulation alone, the percentage of cytoskeletal fibers oriented in each defined sector was evenly distributed, and did not exceed 25% in any 30° sector, which was comparable to the unstimulated configuration (control). However, in devices that were mechanically stimulated with a 7% strain, cells oriented perpendicularly to the strain direction, namely in the 0–30° and 150–180° sectors, with ~30% of the cytoskeletal fibers in each of these sectors. Furthermore, this cellular reorientation was increased when mechanical stimulation at 7% strain was combined with electrical stimulation, resulting in more than 75% of the cytoskeletal fibers becoming aligned within ± 30° of the perpendicular direction.

**Figure 5:**
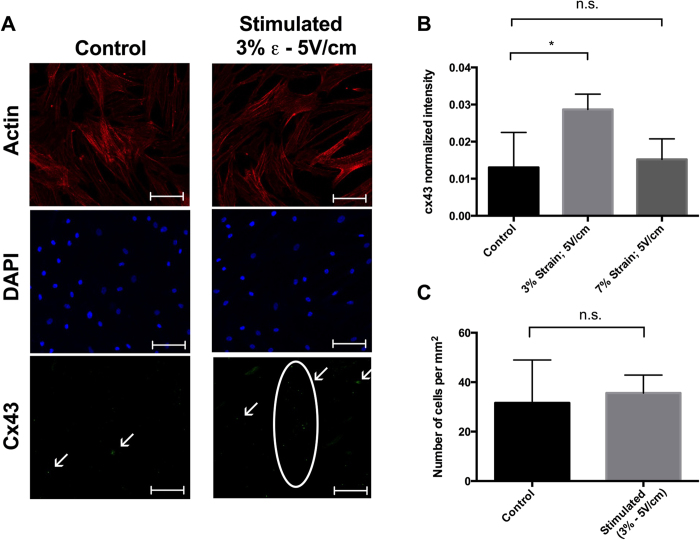
Immunofluorescence data. **(A**) Actin cytoskeleton, nuclei, and CX43 expression in control and electromechanically stimulated devices at 3% strain and 5 V/cm electrical stimulation. Scale bar is 100 μm. (**B**) Quantification and statistical analysis of the CX43 fluorescence intensity under three different conditions: control, 3% strain with 5 V/cm electrical stimulation, and 7% strain with 5 V/cm electrical stimulation. Data are expressed as means ± SD. Two-tailed t-test, *p < 0.05. (**C**) Cell density after 14 days of culture in control and stimulated devices (3% strain and 5 V/cm). Data are expressed as means ± SD.

In mature cardiac cells, the CX43 gap junction protein is essential for intercellular communication when single cells constitute a functional pluricellular complex. Therefore, CX43 serves as a marker of induced differentiation^37^, and its expression was evaluated here by fluorescence immunostaining (Fig. 5A). There were statistically significant differences in CX43 intensities between control samples and cells that underwent electrical stimulation (5 V/cm) coupled with low strain levels (3%) (Fig. 5B). No significant differences were observed between control cells and cells exposed to electromechanical stimulation with a higher strain level (7%). Moreover, no differences in cell density were observed between the stimulated (3% strain and 5 V/cm) and control samples after 14 days in culture (Fig. 5C).

### hMSC gene expression analysis

**Figure 6:**
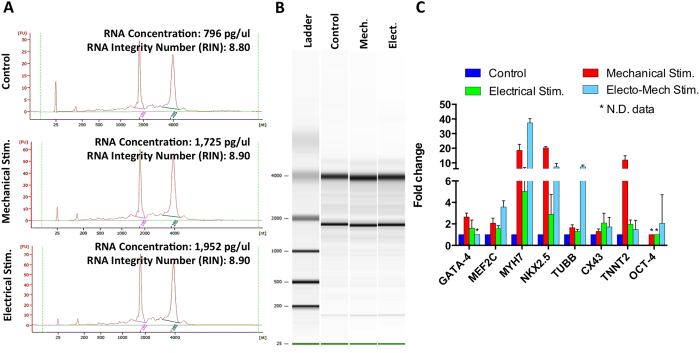
Gene expression analysis. **A**) Assessment of RNA qualities and concentrations using the Agilent Bioanalyzer RNA 6000 Pico Kit. RNA concentrations showed in the electropherograms varied from ~800 pg/μl to ~2 ng/μl, with an integrity number of 8.9 ± 0.1 representing an high quality RNA. (**B**) Agilent Bioanalyzer gel image of total RNA. (**C**) Histogram showing fold-changes in the expression of the selected genes following mechanical stimulation (7% strain), electrical stimulations (5 V/cm) and electromechanical stimulation (strain 7%, electrical signal 5 V/cm) compared to a control configuration without stimulation. GAPDH was chosen as housekeeping gene. Data are expressed as means ± SD.

As a proof-of-principle, relative RNA expression analysis was performed using cells harvested from a single microfluidic device. RNA was extracted from five devices per configuration (*control*, *mechanical and electrical*), and prior to performing quantitative real time polymerase chain reactions (qRT-PCR), the quality and concentration of extracted RNA samples were evaluated with an Agilent Bioanalyzer 2100 (Agilent Technologies, Santa Clara CA, USA) (Fig. 6A,B). To demonstrate the ability to measure changes in gene expression, we identified primers (see Supplementary Table 1) for the specific cardiac markers (*GATA4, MEF2C, MYH7, NKX2.5, TUBB, CX43, TNNT2,* and *OCT4*) using Primer Express 3.0 (Life Technologies), Carlsbad, CA) and optimized their measurement according to a previously reported protocol^38^. GAPDH was chosen as the housekeeping gene. Assays were performed in triplicate for each sample, and the data were analyzed and statistically evaluated using the delta-delta C_T_ method, as previously described^39^.

After 14 days in culture, hMSC RNA concentrations were found to vary from ~800 pg/μl to ~2 ng/μl (Fig. 6A). The results exhibited intact RNA without partial degradation or contaminating genomic DNA, as well as typical 18 S and 28 S peaks, which had integrity numbers of 8.9 ± 0.1 (Fig. 6A). The relative fold-changes in gene expression for mechanically, electrically and electromechanically-stimulated cells were presented (Fig. 6C). Changes in gene expression for GATA4, MEF2C, and TUBB were marginally higher following mechanical stimulation compared to electrical stimulation, with no statistically significant differences observed. Differences between mechanical and electrical stimulation were statistically significant for MYH7, NKX2.5, and TNNT2 expression (p < 0.0001), where mechanical stimulation seems to play a key role in inducing their expression. Only CX43 exhibited a higher fold-change following electrical stimulation consistent with previous qPCR data on MSCs electrically stimulated in a tissue culture plate^22^. Moreover, fold-changes were significantly higher following electromechanical stimulation compared to each single stimulation for MEF2C (p < 0.005), MYH7 (p < 0.0001), NKX2.5 (p < 0.05), and TUBB (p < 0.0001), while TNNT2 was significantly lower (p < 0.0001) following electromechanical stimulation compared to mechanical stimulation but not statistically different compared to electrical stimulation.

## Discussion

The novel micro-scale cell stimulator presented in this study is capable of providing controlled and simultaneous electrical, mechanical, and biochemical stimulations to cells cultured in a microfluidic system. The main advantage of our platform is the ability to apply each stimulation independently or to combine three different stimulations to study interactions of multiple stimuli, which more closely represents complex *in vivo* conditions. We designed the microfluidic device presented herein to accomplish these challenging tasks, and optimizing the geometrical parameters by FEM analysis before production. In addition, each stimulation can be appropriately fine-tuned to achieve specific experimental requirements, offering a wide range of practical bioengineering applications.

We achieved a high level of versatility in stimulating cells on a chip by designing a multilayer PDMS device (Fig. 1), where each layer performs a specific function. Specifically, the pneumatic layer performs mechanical stretching of the cell culture substrate, the conductive layer is used to apply a uniform electric field to cultured cells, and the fluidic layer provides the opportunity to deliver biochemical stimulation. The strain values applied fell within the range of those used in previous macro-scale mechanobiological experiments^32^,^33^. Prior calibration enables the operator to impose a specified strain by simply regulating the amplitude of vacuum pressure applied to the air channels. Values of electrical field gradients of 5 V/cm are easily attainable, and are comparable to those found *in vivo*^40^. The presence of easily-accessible channels for the addition of different media allow for time-varying administrations of factors, as needed, to optimize differentiation.

Our microfluidic device is compatible with fluorescence immunostaining and confocal imaging. Moreover, we demonstrated that our platform can be applied for monitoring spatiotemporal conditions and probing the effects of various stimuli on cells. Of notable interest, an immediate practical application of the device is the electromechanical stimulation of hMSCs, where we observed that mechanical stimulation induced morphological changes in cells and actin cytoskeletal rearrangements in the direction perpendicular to the applied strain (Fig. 4A). Most importantly, the stimulated cells can be easily harvested from the device to perform standard molecular biology analyses, such as qRT-PCR. As result, we observed changes in gene expression generally consistent with our expectation that either mechanical or electrical stimulation helped to induce activation of cardiac myocyte markers (Fig. 6C). When we compare the relative effects of different modes of stimulation, we note similar tendencies, although a stronger effect associated with mechanical strain as compared to electrical stimulation. These methods could be applied to ranges of conditions for both modes of stimulation in order to further investigate the relative impact and the potential for synergistic effects on cell differentiation. These findings intend to show the capabilities of our novel technology that, when combining multiple cell stimulations, offers an important tool for improving investigations of cellular responses in several biological areas, such as stem cell differentiation, cardiac tissue engineering, and regenerative medicine.

## Methods

### Device design and production

The device is comprised of three main layers of polydimethylsiloxane (PDMS) obtained from a base of pre-polymer (Sylgard 184, Dow Corning, Midland, MI, USA) at a ratio of 10:1 base to curing agent. The PDMS layers were obtained by replica molding from corresponding silicon molds produced in a clean room environment using standard photolithography techniques with SU-8 photoresist (MicroChem, Newton, MA, USA), starting from 4′′ silicon wafers. Each mold was initially treated with 50 μl of trichloro(1H,1H,2H,2H-perfluorooctyl)silane (Sigma-Aldrich, St. Louis, MO, USA) under a vacuum for 2 h to facilitate subsequent PDMS demolding. The assembly process of the micro-bioreactor is shown in Fig. 2A(a–f).

Mold A, containing the electrical paths (Fig. 2Aa), was used to cast a CNT-PDMS mixture (20%, w/w) prepared as described in our previous work^41^. The conductive CNT-PDMS mixture was added to fully cover the top of the mold and then carefully spread with a rubber spatula to fill the electrode features. Excess material was removed with a cell scraper (Fig. 2Ab), thus allowing the conductive polymer to occupy only the dedicated vertical features. The mixture was cured by placing the mold into an oven at 80 °C for 4 h, creating the conductive layer for electrical stimulations. Furthermore, a 100 μm thick layer of PDMS was spin-coated on top of mold A (Fig. 2Ac) and allowed to cure in an oven at 80 °C for 4 h to produce a deformable membrane for mechanical stimulations.

Subsequently, the pneumatic actuation and fluidic layers were cast by pouring liquid PDMS on top of mold B and mold C, respectively. After complete polymerization (at 80 °C for 4 h), the pneumatic actuation was removed from the mold and bonded to mold A (Fig. 2Ad) by means of a standard plasma treatment of both layers. The binding is performed with the aid of a microscope to accurately align the pneumatic layer and mold A based on the corresponding features. Individual devices were trimmed from the wafer-size assemblies by cutting out rectangular sections (about 35 × 15 mm) with a razor blade (Fig. 2Ae), and holes were created with biopsy punches (Ted Pella Inc., Redding, CA, USA) to create access ports (1 mm and 3 mm in diameter for pneumatic actuation and cell conditioning, respectively). Each device was plasma bonded to the fluidic layer precisely aligning the features under the microscope (Fig. 2Af). Finally, another plasma treatment allows the bonding of the device onto a glass microscope slide as a supporting substrate. The final device featured two identical central chambers (1.2 mm wide, 15 mm long and 250 μm high), arranged vertically and separated by a thin membrane where the cells were cultured (Fig. 1B,C).

### Finite element modeling (FEM)

A computational model of the microfluidic platform was built to assess the magnitude of the strain imposed on cell monolayers when a negative pressure load was applied to the actuation (side) channels. The system was idealized as a 2-dimensional (2D) model, with all cell, air and media compartments represented, surrounded by a 500 μm thick PDMS frame. The corresponding 2D plane strain finite element model (FEM) was implemented in the Abaqus/Standard 6.10-1 commercial software package (Dassault Systemes Simulia Corp., Providence, RI, USA), and discretized with 8-node biquadratic plane strain quadrilateral (CPE8) elements with a mapped meshing scheme. Mesh sensitivity studies were conducted to ensure consistency of results in terms of nodal displacement of the cell culture membranes. The resulting elements had an average edge size of ~25 μm, thus generating a final mesh of ~10928 elements. As the mesh density increased, the displacement of the chosen nodes varied by less than 1%.

Different material properties were assigned to different parts of the geometry, depending on the corresponding load conditions^42^. In particular, the nonlinear behavior of PDMS was considered by implementing a hyperelastic model, where high deformations were present (i.e. side membranes). The stress-strain relationship of a hyperelastic material is represented by the following formula:


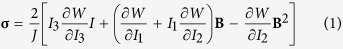


where **σ** is the Cauchy stress, *W* is the strain energy function, **B** is the left Cauchy-Green deformation tensor, *J* is the volume ratio, and *I*_1_, *I*_*2*_, and *I*_3_ are the first, second, and third invariants of **B**, respectively. For a Mooney-Rivlin material, such as PDMS, the strain energy function, *W*, can be described as:


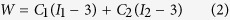


where *C*_*1*_ and *C*_*2*_ are material constants. The material constants were set as being equal to *C*_*1*_ = 254 kPa and *C*_*2*_ = 146 kPa^43^.

Conversely, where small deformations were assumed, the PDMS was modeled as being incompressible and linearly elastic and the spin-coated membrane was modeled as being stiffer than the bulk material^44^. The material properties, Young’s modulus, and Poisson ratio were thus set as being equal to *E* = 0.60 MPa and *ν* = 0.5 for the bulk material, and *E* = 1.2 MPa and *ν* = 0.5 for the membrane, respectively. The modeled geometry is depicted in Supplementary Fig. 1, highlighting the material properties based on the assigned sections.

The boundary conditions for the FEM were as follows: (i) the edges of the outer PDMS frame were fixed (encastre constrain) to consider their continuity with the bulk material of the device; (ii) self-contact was set, among all the internal surfaces of the compartments, with a no-slip tangential behavior once surfaces came into contact; (iii) a static pressure of −700 mmHg was applied to the inner surfaces of the vacuum compartment (orange in Supplementary Fig. 1), through 20 incremental steps.

### Device characterization

Device characterization was focused on two aspects, namely membrane deformation as a function of negative pressure (vacuum) and electric signal recording within the cell culture channel.

For strain characterization, iron particles (Inframat Advanced Materials, Manchester, CT, USA) with an average diameter of 1 μm were suspended in the PDMS mixture preparation and mixed for 1 min to break up aggregates. In this way, it was possible to embed the particles in the spin-coated membrane and track their displacement (Fig. 3A,B). A range of actuation pressures, ranging from 0 mmHg to −700 mmHg (steps of −50 mmHg), was applied to the pneumatic layer using an eccentric diaphragm vacuum pump (Trivac D&B, TX, USA). Pictures were taken at various actuation conditions using a 200X USB Microscope (AnMo Electronics Corporation, Taiwan) and stored for subsequent image processing.

A custom-written MATLAB code (MathWorks, Natick, MA, USA) was used to convert the images in binary format and track 25 bead locations at the vertices of a 5 × 5 matrix, using the particle-tracking algorithm of ImageJ^45^ plugin (Speckle TrackerJ^30^). As a result, the X and Y positions of each selected particle spot for each video frame were saved in a dataset file. The dataset files were then analyzed in order to visualize the strain map in different directions (XX, YY, and XY). This procedure was repeated for ten different devices and considering different membrane regions in the same device.

For the electrical characterization, a metal needle was injected into the PDMS from the top layer in the center of the cell seeding area, and the signal was recorded with an oscilloscope.

After production, each device was checked for mechanical and electrical functionality by controlling leakages during vacuum application and monitoring signals during electrical stimulation.

### Control system

The system illustrated in Fig. 2B was assembled to perform cyclic-controlled vacuum and electrical stimulation procedures. A glass reservoir, connected to a diaphragm vacuum pump (Cole Parmer, Vernon Hills, IL, USA), was used as a vacuum source. An electronically-controlled valve (ITV Electronic Vacuum Regulator, SMC, Noblesville, IN, USA), controlled by a frequency generator, enabled tuning of the output pressure signal by imposing switching conditions between vacuum source and atmospheric pressure (both in terms of frequency and duty cycle). Furthermore, an electrical stimulator (STG4002, Multichannel Systems, Reutlingen, BW, Germany) was used to deliver a bipolar electrical signal, synchronized with the mechanical actuation, where negative-to-positive variation in the slope of the frequency generator signal was used as a trigger. Each device was equipped with tubing for pneumatic actuation and with gold pin wires for electrical signals (Fig. 2C). A manifold for pneumatic distribution and electrical connectors for signal distribution were placed inside the incubator and connected through the back port of the incubator to the output line of the valve and the output connectors of the electrical stimulator.

### Cell culture and stimulation parameters

Before seeding cells into the microfluidic chambers, devices were sterilized by autoclaving and drying in an oven (80 °C) overnight, followed by incubation with 50 μg/ml human fibronectin (Life Technologies) for 30 min (37 °C, 5% CO_2_). Human bone marrow-derived MSCs (hMSC, Lonza, Basel, Switzerland) were seeded on fibronectin-coated T75 tissue culture flasks and cultured with growth medium (DMEM, 10% FBS, and 1% penicillin/streptomycin, Life Technologies). Upon reaching 80% confluence, cells were trypsinized (0.05% Trypsin-EDTA (1x), phenol red, Life Technologies) and resuspended in fresh growth medium at a density of 1.5 × 10^6^ cells/ml. Aliquots of 20 μl cell suspensions were used for seeding microfluidic devices by injecting the suspensions into a fluidic well with conventional pipettes. To allow adequate adhesion of cells to the cell culture membrane, electrical and/or mechanical stimulation was applied at 24 h after seeding, and cells were maintained in culture in DMEM for an additional 14 days.

Mechanical stimulation was set as strain of 3% or 7% with 1 Hz frequency, following literature reporting a cyclic uniaxial stretch of 5–10% and 1–2 Hz for artificial cardiac tissue^3^,^46^,^47^. For the electrical stimulation we selected a pulsed stimulation to replicate the electrical environment of human heart tissue^21^. In particular, we chose biphasic square pulses of 1 ms at 1 Hz as biphasic stimulations are physiologically relevant and are known to avoid cell damage^48^,^49^. A field in the range of 5 V/cm was selected because it falls within the range of physiological values, 0.1–10 V/cm^18^, and is consistent with several previous studies^50^,^51^,^52^,^53^.

### Quantification of cell orientation

Cell orientation analysis was performed using Fiji software (Directionality plugin)^34^ after 14 days of stimulation. The analysis was carried on cytoskeleton filaments, using images acquired after counterstaining. Five devices for each configuration (control, mechanical stimulation, or electrical stimulation, and combinations thereof) were analyzed. Statistical analysis of the means was performed by a two-tailed t-test, and differences were considered statistically significant in cases where the level of confidence exceeded 95%.

### Immunofluorescence staining and intensity quantification

To demonstrate the compatibility of the devices with fluorescent microscopy techniques, cells were stained following standard immunofluorescence protocols. After washing cells with 1x PBS, cells were fixed with 4% paraformaldehyde for 10 min and washed twice in 1x PBS. A solution of 0.1% Triton-X100 was used to permeabilize the cells for 15 min (Image-iT^®^ Fixation⁄Permeabilization Kit, Life Technologies). To prevent non-specific antibody binding, cells were treated for 2 h with a blocking buffer containing 1x PBS, 4% goat serum, and 5% bovine serum albumin. Samples were then incubated overnight at 4 °C with rabbit polyclonal antibody (Abcam, Cambridge, UK) against CX43 at a 1:200 dilution. After three washing steps with washing buffer, WB (1x PBS, 1% bovine serum albumin), cells were incubated with an Alexa Flour^®^ 488 secondary antibody (Life Technologies) at a 1:200 dilution for 1 h in the dark and washed again with WB. Finally, counterstaining was performed using DAPI (Sigma-Aldrich) for nuclei at a 1:200 dilution and ActinRed™ 555 ReadyProbes® Reagent (Life Technologies) for actin cytoskeleton staining. Images were captured using a LSM-780 confocal microscope (Zeiss, Oberkochen, Germany) and processed with Imaris software (Bitplane Scientific Software, Zurich, Switzerland). CX43 expression was quantified from immunofluorescence images through the ImageJ software, for at least three regions of interest (ROIs) for each device. Five devices were considered for each condition. The fluorescence intensities were normalized by the number of cells in the ROIs and plotted as mean ± SD. Cell number per mm^2^ was assessed by counting the nuclei stained with DAPI and dividing by the area of the ROI. Statistical analysis was performed by a two-tailed t-test.

### qRT-PCR

Quantitative real time polymerase chain reactions (qRT-PCR) were performed for a range of cardiac markers, using the StepOnePlus Real-Time PCR System (AB Applied Biosystems, Life Technologies). Cells were trypsinized within the device and collected in a 1 ml Eppendorf vial through three washing steps. RNA extraction was performed with the PicoPure® RNA Isolation Kit (Life Technologies), following the manufacturer’s recommendations, including the additional DNAse treatment step proposed by the manufacturer. qRT-PCR was carried out with RNA from cells harvested from a single microfluidic device. Five devices for each configuration (control, electrical stimulation, mechanical stimulation and electromechanical stimulation) were considered for the RNA extractions.

The qualities and concentrations of the extracted RNA samples were evaluated with the Agilent Bioanalyzer 2100 (Fig. 6A,B). To perform qRT-PCR, RNA (1 ng) was reverse transcribed into cDNA using the Sensiscript Kit (Qiagen, Venlo, Limburg, Netherlands). Polymerase chain reactions were performed using Power SYBR Green (Power SYBR® Green PCR Master Mix, Life Technologies) following the manufacturer’s recommended protocol in 20 μl reaction volumes. Specific primers for amplifying GATA4, MEF2C, MYH7, NKX2.5, TUBB, CX43, TNNT2, and OCT4 mRNA are shown in Table 1 (Supplemental materials). Primers were designed using Primer Express 3.0 (Life Technologies) and optimized as described previously^38^. GAPDH was chosen as the housekeeping gene. The final reaction mixture for each well consisted of 10 μl of Power SYBR Green, 0.5 μl of cDNA, 8.5 μl of water, and 1 μl of forward and reverse primers. Reaction conditions were 95 °C for 20 s, followed by 40 cycles of denaturation at 95 °C for 3 s, annealing/extension for 30 s at 60 °C, and data collection for 3 s at 60 °C. The data obtained by qRT-PCR were then analyzed and statistically evaluated using the delta-delta Ct method^38^.

## Additional Information

**How to cite this article**: Pavesi, A. *et al.* Controlled electromechanical cell stimulation on-a-chip. *Sci. Rep.* **5**, 11800; doi: 10.1038/srep11800 (2015).

## Supplementary Material

Supplementary information
