# Supplementary material for: Controlled electromechanical cell stimulation on-a-chip
Source: Sci Rep. 2015 Jul 2;5:11800. doi: 10.1038/srep11800 (PMC4488866; doi:10.1038/srep11800)
Supplement: Supplementary information [file srep11800-s1.doc]

**Supplementary material**

**Controlled electromechanical cell stimulation on-a-chip**

**Andrea Pavesi**^1^, **Giulia Adriani**^1^, **Marco Rasponi**^2^, **Ioannis K. Zervantonakis**^3^, **Gianfranco B. Fiore**^2^, and **Roger D. Kamm**^1, 4, *^

^1^Biosym IRG, Singapore-MIT Alliance for Research and Technology, Singapore

^2^Department of Electronics, Information and Bioengineering, Politecnico di Milano, Milano, Italy

^3^Department of Cell Biology, Harvard Medical School, USA

^4^Departement of Biological Engineering Massachusetts Institute of Technology, USA

***** Corresponding author rdkamm@mit.edu


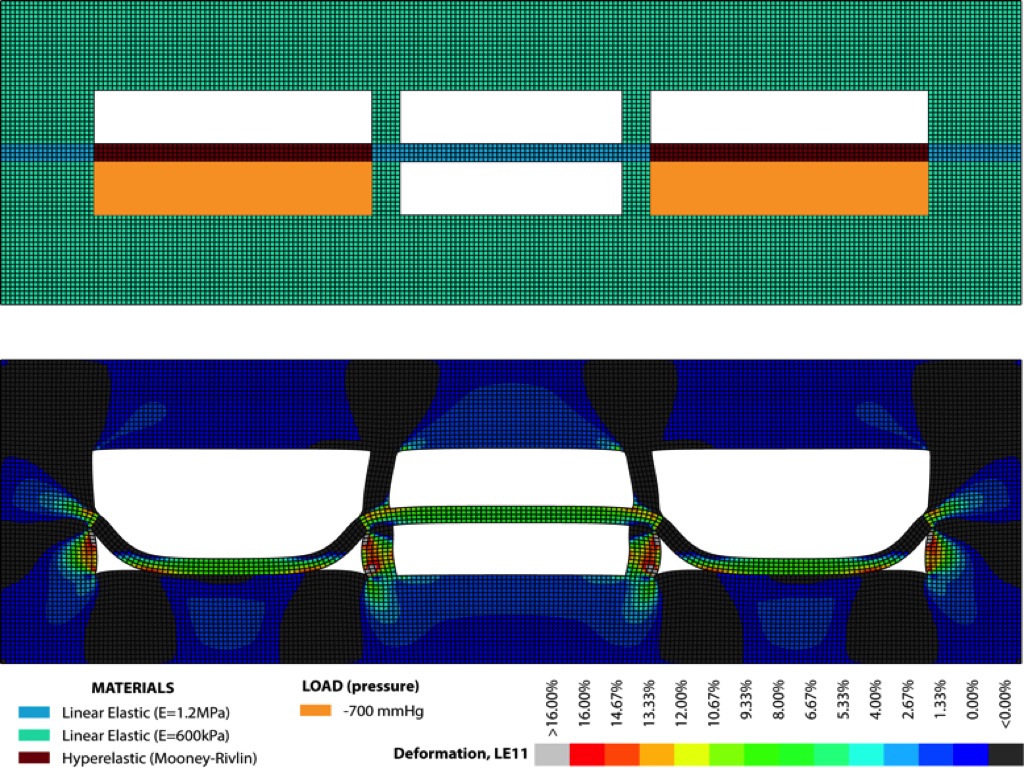


Supplementary Figure 1. Two-dimensional model of the device cross-section with lateral membranes. a) Unstretched configuration. Vacuum compartments are shown in orange. b) Stretched configuration when a static pressure of −700 mm Hg was applied to the vacuum compartments. The system was constituted by PDMS with different deformation regimes, namely, incompressible and linear elastic for small deformations, or hyperelastic for larger deformations.

| **Gene** | **Forward primers (5′ to 3′)** | **Reverse primers (5′ to 3′)** |
| --- | --- | --- |
| ***GAPDH* (Housekeeping gene)** | ATGGAAATCCCATCACCATCTT | CGCCCCACTTGATTTTGG |
| ***GATA4*** | TTTCCCCTTTGATTTTTGATCTTC | AACGACGGCAACAACGATAAT |
| ***MEF2C*** | GCTGAGCGTGCTGTGTGACT | AGCTTGTTGGTGCTGTTGAAGA |
| ***MYH7*** | TTCGTGCCTGATGACAAACAG | CTCGGTCTCGGCAGTGACTT |
| ***NKX2.5*** | AGGACCCTAGAGCCGAAAAG | CTTGCACTTGTAGCGCCG |
| ***TUBB*** | AGGCCTTCCTCCACTGGTACA | AGCCTCGGTGAACTCCATCTC |
| ***CX43*** | CAAGTGAGCAAAACTGGGCTAA | CGCCTGCCCCATTCG |
| ***TNNT2*** | CCGGGCGCTGGAAATAG | CGAGCGAGGAGCAGATCTTT |
| ***OCT4*** | CGACCATCTGCCGCTTTG | GCCGCAGCTTACACATGTTCT |

**Supplementary Table 1.** Primers used for qRT-PCR
